# Dichotomous Dopaminergic Control of Ventral Pallidum Neurons

**DOI:** 10.3389/fncel.2018.00260

**Published:** 2018-08-22

**Authors:** Martin Clark, Enrico Bracci

**Affiliations:** Department of Psychology, The University of Sheffield, Sheffield, United Kingdom

**Keywords:** ventral pallidum, dopamine, multielectrode, reward, basal ganglia

## Abstract

The ventral pallidum (VP) is crucially involved in reward processing. Dopaminergic afferents reach the VP from the ventral tegmental area (VTA). Recent *in vivo* studies suggest dopamine application increase the firing in the VP. However, little is known about the cellular effects of dopamine within the VP. We aimed to address this paucity of data using brain slices containing the VP and multi-electrode array recordings. Dopamine significantly affected firing in 86% of spontaneously active VP neurons. Among the affected neurons, 84% were excited, while 16% were inhibited. The selective D1-like receptor agonist SKF81297 also had modulatory effects on the majority of VP neurons, but its effects were universally excitatory. On the other hand, the D2-like receptor agonist quinpirole had modulatory effects on 87% of VP neurons studied. It caused significant inhibitory effects in 33% of the cases and excitatory effects in the remaining 67%. The effects of D1-like receptor activation were presynaptic as blocking synaptic transmission with low Ca^2+^ abolished the effects of SKF81297 application. Furthermore, SKF81297 effects were abolished by blocking ionotropic glutamate receptors, suggesting that D1-like receptors boost glutamate release, which in turn excites VP neurons through postsynaptic glutamate receptors. Effects caused by D2-like receptor activation were found to involve pre and postsynaptic mechanisms, as low Ca^2+^ abolished the excitatory effects of quinpirole but not the inhibitory ones. Increases in firing frequency (ff) to quinpirole application were abolished by a group 2/3 mGluR antagonist, suggesting that D2-like receptors cause presynaptic inhibition of glutamate release, resulting in reduced postsynaptic activation of inhibitory mGluRs. Conversely, the inhibitory effects of quinpirole persisted in low Ca^2+^ and therefore can be attributed to postsynaptic D2-like receptor activation. VP neurons excited by dopamine had shorter spike half-widths and are excited by D1-like receptors (presynaptically) and by D2-like receptors (postsynaptically). VP neurons inhibited by dopamine have longer spike half-widths and while D1-like receptor activation has a presynaptic excitatory influence on them, D2-like receptor activation has a postsynaptic inhibitory effect that prevails, on balance. These data provide novel insights into the cellular mechanisms by which dopamine controls information processing within the VP.

## Introduction

The ventral pallidum (VP) is a key output structure for the ventral striatum (Smith et al., 2009). It also forms multiple feedback loops with some of the key structures involved in reward signaling, including the nucleus accumbens (NAc), the medial pre-frontal cortex, the basal lateral amygdala, the subthalamic nucleus and the ventral tegmental area (VTA; Root et al., 2015). The VP is known to have populations of GABAergic interneurons and cholinergic neurons that belong to the forebrain magnocellular cholinergic system (Gritti et al., 1993; Pang et al., 1998; Bengtson and Osborne, 2000).

Interest in the VP has increased recently as the VP, and its connective circuitry, is heavily associated with reward, aspects of reinforcement and motivational salience (Cromwell and Berridge, 1993; Itoga et al., 2016; Richard et al., 2016). Indeed Smith et al. (2009) conclude that it is an essential integrative region for reward and reward learning and may well be a “limbic final common pathway.” The VP also plays a key role in addictive behavior towards drugs of abuse (Hubner and Koob, 1990; Hiroi and White, 1993; Robledo and Koob, 1993; Gong et al., 1996; Fletcher et al., 1998; Tang et al., 2005).

The VP has a substantial dopaminergic projection arising from the VTA (Klitenick et al., 1992; Smith and Kieval, 2000; Root et al., 2015) and dopamine receptor subtypes (D1, D2, D3) are known to be expressed within the VP (Mansour et al., 1990; Richtand et al., 1995). However, little is known about the effects of dopamine in the VP. Heidenreich et al. (1995) have shown that, *in vivo*, D1-like receptor agonists increase the firing frequency (ff) in approximately 45% of VP neurons. Napier and Maslowski-Cobuzzi (1994) found that D2-like receptor agonists also modulated the firing frequency in the VP *in vivo* and Johnson and Napier (1997) suggested that dopamine effects depend on modulation of GABA inputs into VP. However, the cellular mechanisms activated by dopamine in the VP are still obscure. *In vivo* experiments are not ideal to clarify this issue, as the concentrations of ligands cannot be precisely controlled and because the presence of multiple active inputs to both the VP and the dopaminergic neurons in the VTA, greatly complicate the interpretation of the results.

Elucidating the cellular mechanisms underlying the dopamine action in the VP is an extremely important aim, as there is a strong connection between dopamine and the role of the VP in reward and addictive behavior. Recently, manipulation of dopamine in the VP has been shown to dramatically affect avoidance learning. Péczely et al. (2014) and Lénárd et al. (2017) have shown that D1-like and D2-like receptors within the VP are involved in the formation and retention of avoidance learning. Further, research (Maslowski and Napier, 1991b; Stout et al., 2016) has illustrated the role of dopamine in the VP on the effects of drugs of abuse. Creed et al. (2016) have suggested that targeted research on the VP is essential as it may provide novel strategies to treat addiction/addictive disorders.

## Materials and Methods

### Animals

This study was carried out in accordance with the recommendations of the UK Home Office’s Animals (Scientific Procedures) Act 1986. The protocol was approved by the local Sheffield University Ethics Committee. Extracellular *in vitro* recordings were obtained from C57 mice. In some instance, due to availability, mice from the transgenic line ChR2-NNos (Jackson Laboratory, Bar Harbor, ME, USA) were used. No differences were found in the electrophysiological activity of VP neurons between these animals and the C57 mice.

### Slice Preparation

Mice aged between 28 days and 42 days were killed by cervical dislocation and death confirmed by decapitation. The brain was rapidly removed from the skull and parasagittal slices of 400 μm obtained using a vibroslicer (Cambden Instruments) immersed in ice cold (5°C), oxygenated (saturated 95% O_2_ and 5% CO_2_) sucrose cutting solution. This solution was made up fresh daily and contained (in mM): Sucrose (184), KCl (2.5), NaH_2_PO_4_ (1.2), NaHCO_3_ (30), HEPES (20), Glucose (25), sodium ascorbate (5), Thiourea (2), sodium pyruvate (3), MgSO_4_.7H_2_O (10), CaCl_2_.2H_2_O (0.5).

Once cut, slices were immediately transferred to a recovery chamber maintained at 26°C, containing a Tris recovery solution, which was continuously aerated with a carbogen mixture of 95% O_2_ and 5% CO_2_ gas. The Tris recovery solution was made up fresh daily and contained (in mM): Tris HCl (76), Tris base (19.5), KCl (2.5), NaH_2_PO_4_ (1.2), NaHCO_3_ (30), HEPES (20), Glucose (25), sodium ascorbate (5), Thiourea (2), sodium pyruvate (3), MgSO_4_.7H_2_O (10), CaCl_2_.2H_2_O (0.5). The slices remained in this chamber for 30 min, before being transferred to another chamber for storage. This chamber was also maintained at 26° C and contained standard aCSF, which was continuously aerated with a carbogen mixture of 95% O_2_ and 5% CO_2_ gas. The standard aCSF was also made up fresh daily and contained (in mM): NaCl (124), KCl (3), NaH_2_PO_4_ (1.2), NaHCO_3_ (26), Glucose (15), MgSO_4_ (2), CaCl_2_ (2). The slices were then left for a minimum of 60 min to equilibrate and recover before electrophysiological recordings commenced.

### pMEA (Perforated Multi-Electrode Array) Electrophysiological Recordings

Neural network activity was monitored and recorded using a perforated multi-electrode array (pMEA, Multi-Channel Systems, Reutlingen, Germany). The pMEA contained 60 embedded electrodes constructed of titanium nitrite. Each of these electrodes has a diameter of 30 μm and they are spaced at 200 μm. Recorded electrical activity for selected channels of interest was digitized at a sampling rate of 10 kHz using a MEA1060-Up-BC amplifier and MC_Rack (version: 4.6.2) software (Multi-Channel Systems, Reutlingen, Germany).

For recording slices were transferred to the pMEA chamber, already in position in the amplifier. Once the slice was moved into position over the electrodes on the MEA, the bottom flow was switched on to produce suction and fix the slice into position. A mesh harp was then placed on top of the slice and a top flow applied as quickly as possible to maintain healthy slices. Both the top and bottom flow (perfusion) contained continuously aerated aCSF. The bottom flow rate was maintained at 0.65–1 ml/min and the top flow was maintained at 3–5 ml/min. Activity was monitored for 1 h before recordings commenced.

Depending upon the experimental protocol, two types of aCSF were used to perfuse the slice once in the MEA recording chamber. One referred to as standard aCSF/control and a second with low Ca^2+^ levels referred to as low Ca^2+^ aCSF. The standard aCSF contained (in mM): NaCl (124), KCl (3), NaH_2_PO_4_ (1.2), NaHCO_3_ (26), Glucose (15), MgSO_4_.7H_2_O (2), CaCl_2_.2H_2_O (2). The low Ca^2+^ aCSF was the same as the “standard” aCSF, bar a reduced quantity of CaCl_2_ (0.2 mM).

### Slice Visualization

To identify the correct area of the mouse slice for recording of the VP neurons, the pMEA was placed in the amplifier and then under an Olympus BX51 microscope, with a 4× lens. The slice was viewed via a Tucson digital microscope camera, which was sending a live feed to a Viglen computer (4 gb of memory and an i5 processor) running IS capture software.

### Pharmacology

All drugs were obtained from either Tocris Biosciences (UK) or Sigma Aldrich and were bath applied into the header reservoir feeding the top perfusion flow of the pMEA at the following concentrations:

1. 4a*R*-trans)-4,4a,5,6,7,8,8a,9-Octahydro-5-propyl-1*H*-pyrazolo [3,4-*g*]quinoline hydrochloride: (-)-Quinpirole hydrochloride (quinpirole), 20 μM.
2. (±)-6-Chloro-2,3,4,5-tetrahydro-1-phenyl-1*H*-3-benzazepine hydrobromide: (SKF81297), 20 μM.
3. (*S*)-(-)-5-Aminosulfonyl-*N*-[(1-ethyl-2-pyrrolidinyl)methyl]-2-methoxybenzamide (sulpiride), 20 μM.
4. (6a*S*-*trans*)-11-Chloro-6, 6a, 7, 8, 9, 13b-hexahydro-7-methyl-5*H*-benzo[*d*]naphth[2,1-*b*]azepin-12-olhydrobromide (SCH39166), 20 μM.
5. 2,3-dihydroxy-6-nitro-7-sulfamoyl-benzo[f]quinoxaline-2, 3-dione (NBQX), 20 μM.
6. D-(-)-2-Amino-5-phosphonopentanoic acid (AP5), 20 μM.
7. (*RS*)-α-Methyl-4-carboxyphenylglycine (MCPG), 20 μM.
8. (*RS*)-α-Methyl-4-sulfonophenylglycine (MSPG), 10 μM.
9. 3, 4-Dihydroxyphenethylamine hydrochloride: (dopamine), 30 μM.

Washout of any pharmacological treatment was considered complete when firing frequency recorded over a period of 1,200 s was not significantly different from that observed over a 1,200 s period just before drug application.

### Data Analysis

Data was acquired using MC_Rack software (version: 4.6.2) and a MEA1060-Up-BC amplifier (Multi-Channel Systems, Reutlingen, Germany). These files (.mcd) were then converted to .ced files using multichannel data manager software (version: 1.9.7, Multi-Channel Systems, Reutlingen, Germany) for off-line analysis using Spike 2 software (C.E.D).

For all recordings, spike sorting was carried out offline using dedicated Spike2 (C.E.D.) software. This software uses an automated waveform matching system to construct waveform templates and allows the user to set an appropriate threshold for detection of individual units. When multiple units were detected in a trace, discrete clustering of waveforms within a template was verified through principal components analysis, also implemented by Spike2.

See figure legends for information on result expression. All error bars are expressed as SEM. In order to assess differences in a neuron’s firing frequency in different pharmacological conditions, we measured consecutive ISIs during the final 1,200 s of each condition. Average ISIs for relevant conditions were then compared using a Student’s *t*-test. A statistically significant difference was considered to be present if *P* < 0.05. If a treatment caused a significant increase in ISI, we refer to this observation in the results as a significant decrease in firing frequency and an inhibitory effect of the treatment. If a treatment caused a significant decrease in ISI, we refer to this observation as a significant increase in firing frequency and an excitatory effect of the treatment.

**Figure 1:**
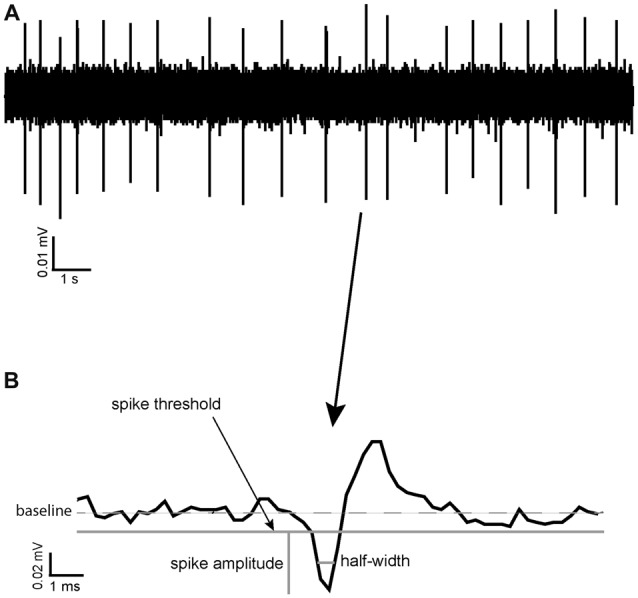
Spike half-width calculation. **(A)** A raw data trace of tonic neuronal activity measured in the ventral pallidum (VP). **(B)** An example of a typical spike recorded in the VP with the reference point used for calculation of its spike half-width, halfway between baseline and maximum negative amplitude.

Coefficient of variation (CoV) was calculated as a measure of spike train variability in different pharmacological conditions and as a potential way of identifying different neuronal types in the VP. It was calculated as standard deviation (ISI)/Mean ISI. Threshold for spike detection was considered to be reached when the recorded voltage departed from baseline (0 mV in AC recording mode) by more than the standard deviation of the voltage recorded for that channel during an apparently quiescent period (of at least 3 s). Spikes consisted of a biphasic negative-positive waveform. Spike amplitude was defined as the difference between the negative voltage peak and the spike threshold level defined above. Spike half-width was defined as the time the value of the recorded voltage (measured from the threshold level) remained more negative than half of the spike amplitude (Figure 1; Pettersen and Einevoll, 2008).

## Results

### Dopamine Application Has Dual Effects on VP Neurons

**Figure 2:**
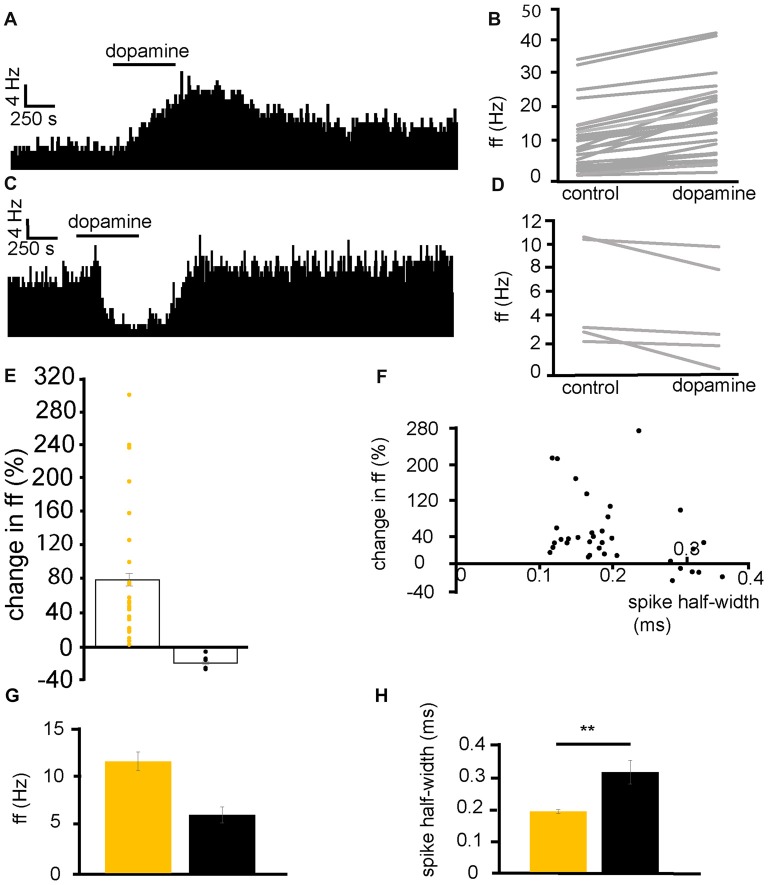
Dopamine application has dual effects on VP neurons. **(A)** Excitatory response to dopamine application in a VP neuron. **(B)** Changes in firing frequency (ff) for 30 VP neurons in experiments similar to that illustrated in panel **(A)**. Firing frequency was measured before and during the application of dopamine. **(C)** Inhibitory response to dopamine application in a VP neuron. **(D)** Changes in firing frequency for five VP neurons in experiments similar to those illustrated in panel **(C)**. Firing frequency was measured before and during the application of dopamine. **(E)** Distribution of excitatory (yellow) and inhibitory (black) responses (% change compared to baseline) to dopamine application in VP neurons with their corresponding average values. **(F)** Spike half-widths (ms) for each neuron compared to % change in firing frequency in response to dopamine application. **(G)** Firing frequency for VP neurons excited by dopamine (yellow) and those inhibited (black). **(H)** Significant differences in spike half-width (ms) for VP neurons excited bydopamine application (yellow) and those inhibited (black). In this and followingfigures, *P < 0.05 and **P < 0.01.

From six experiments, 35 VP neurons were selected for analysis as they responded to dopamine application. 29/35 neurons displayed significant (*P* < 0.05) increases in firing frequency to application of dopamine (Figures 2A,B) while 5/35 displayed significant (*P* < 0.05) decreases in firing frequency (Figures 2C,D). In the neurons that were excited by dopamine, the average increase in firing frequency was 78 ± 14% (Figure 2E). In the neurons that were inhibited by dopamine, the average decrease in firing frequency was −17 ± 3% (Figure 2E).

Baseline firing frequency and spike half-width were calculated for all neurons. Two clusters of neurons, with different spike half-width profiles can be identified in Figure 2F, those with a shorter spike half-width (cluster I) in the range 0.12–0.24 ms and those with a longer spike half-width (cluster II), in the range 0.28–0.36 ms. Those neurons with a shorter spike half-width (cluster I) are all excited by dopamine, while neurons with a longer spike half-width (cluster II) show both excitatory and inhibitory responses to dopamine. The baseline firing frequency of VP neurons excited by dopamine was not significantly different (*P* > 0.05) from that of those inhibited by dopamine (Figure 2G). However, spike half-width was significantly (*P* < 0.05) larger 0.32 ± 0.04 ms in neurons inhibited by dopamine than in those excited by dopamine 0.19 ± 0.01 ms as illustrated in Figure 2H.

We concluded that there are two electrophysiologically distinct populations of VP neurons that could be consistently differentiated by their spike half-widths. Those with a shorter half-width (cluster I) were consistently excited by dopamine, however, for cluster II, some neurons were excited by dopamine while others were inhibited by dopamine.

### Receptors Responsible for Dopamine Effects on VP Neurons

Mansour et al. (1990) found that D1, D2 and D3 receptors are present within the VP. To identify the dopamine receptors involved in excitatory and inhibitory VP neurons responses, we carried out experiments using D1-like and D2-like receptor agonists.

In five experiments, 34 VP neurons were selected for analysis as they responded to application of the D2-like receptor agonist quinpirole. 28/34 of these neurons displayed significant (*P* < 0.05) changes in firing frequency in response to quinpirole application with, 9/34 displaying significant (*P* < 0.05) decreases in firing frequency and 19/34 displaying significant (*P* < 0.05) increases in firing frequency in response to quinpirole application. In a further four experiments, 29 VP neurons were selected for analysis as they responded to application of the D1-like receptor agonist SKF81297. 22/29 of these neurons displayed significant (*P* < 0.05) increases in firing frequency in response to SKF81297 application.

In order to further explore the role of dopamine receptor types, we carried out experiments in which quinpirole and SKF81297 were applied sequentially (SKF81297 was applied after complete washout of quinpirole).

**Figure 3:**
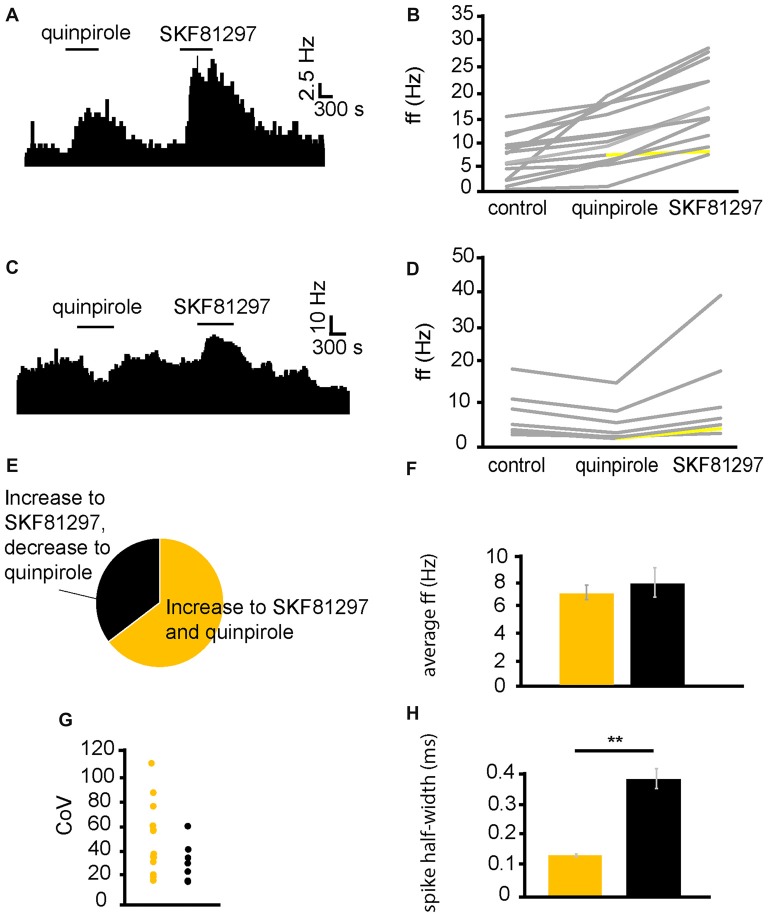
D1-like receptor agonists excite while D2-like receptor agonists excite and inhibit VP neurons. **(A)** Excitatory response to the application of quinpirole and SKF81297 (separated by complete washout out). **(B)** Changes in firing frequency for 14 VP neurons induced by quinpirole application and SKF81297 application similar to that illustrated in panel **(A)**. Firing frequency was measured; before the application of quinpirole, after the application of quinpirole and subsequently, after a period of wash out, for the application of SKF81297. Responses characterized by significant (*P* < 0.05) changes in firing frequency are in gray, the other ones in yellow. **(C)** Inhibitory response to the application of quinpirole and excitatory response to the application of SKF81297 (separated by complete washout). **(D)** Changes in firing frequency for seven VP neurons induced by quinpirole application and SKF81297 application similar to that illustrated in panel **(C)**. Firing frequency was measured; before the application quinpirole, after the application of quinpirole and subsequently, after a period of wash out, for the application of SKF81297. Responses characterized by significant (*P* < 0.05) changes in firing frequency are in gray, the other ones in yellow. **(E)** The majority of neurons analyzed responded with increases in firing frequency in response to both quinpirole and SKF81297 (yellow), but a minority decreased their firing frequency in response to quinpirole and increased their firing frequency in response to SKF81297 (black). **(F)** Firing frequency for those neurons excited by both quinpirole and SKF81297 (yellow) and those neurons that were inhibited by quinpirole (black). **(G)** Coefficient of variation (CoV) for those neurons excited by both quinpirole and SKF81297 (yellow) and those inhibited by quinpirole (black). **(H)** Significant differences in spike half-width (ms) for those neurons excited by both quinpirole and SKF81297 (yellow) and those that were inhibited by quinpirole (black).

From six experiments, 21 VP neurons were identified for analysis as they responded to quinpirole and SKF81297 application. Sequential application of quinpirole and SKF81297 resulted in 13/21 neurons displaying significant (*P* < 0.05) increases in firing frequency to both SKF81297 and quinpirole (Figure 3B). 6/21 neurons displayed significant (*P* < 0.05) decreases in firing frequency to quinpirole and significant (*P* < 0.05) increases, in firing frequency, to SKF81297 (Figure 3D). This means that 62% of the neurons in the VP were excited by both SKF81297 and quinpirole, while 29% of neurons were inhibited by quinpirole but excited by SKF81297 (Figure 3E).

In order to ascertain if the responses to quinpirole and SKF81297 were produced by distinct types of neurons in the VP, baseline firing frequency (Figure 3F), CoV (Figure 3G) and spike half-width (Figure 3H) were measured for neurons that were excited by both quinpirole and SKF81297 and for those excited by SKF81297 but inhibited by quinpirole. Baseline firing frequency and CoV (Figures 3F,G) were not significantly (*P* > 0.05) different in the two populations. On the other hand, there was a significant (*P* < 0.05) differences in spike half-width (Figure 3H). Neurons excited by both quinpirole and SKF81297 had a spike half-width of 0.15 ± 0.08 ms, while those excited by SKF81297 but inhibited by quinpirole had a spike half-width of 0.37 ± 0.03 ms. The range of spike half-widths for these populations was similar to those of cluster I and cluster II (Figure 2F). Cluster I had a spike half-width range of 0.12–0.24 ms (Figure 2F), which compares well to neurons that were excited by both quinpirole and SKF81297 that had a spike half-width range of 0.10–0.22 ms. On the other hand, cluster II had a spike half-width range of 0.28–0.36 ms (Figure 2F), which compared well to neurons that were excited by SKF81297 but inhibited by quinpirole, which had a spike half-width range of 0.27–0.42 ms. This suggests that cluster I in Figure 2F correspond to those neurons excited by both quinpirole and SKF81297 (Figure 3B), while cluster II in Figure 2F correspond to those neurons excited by SKF81297, but inhibited by quinpirole (Figure 3D).

We concluded that D1-like and D2-like receptors can cause, independently, an increase in firing frequency in the majority of VP neurons, but that an electrophysiologically distinct minority of neurons, characterized by longer spike half-width durations, are inhibited by D2-like receptors. Therefore, it appeared likely the effects of dopamine in the neurons inhibited by D2-like receptors is depended on the net balance between the excitatory influence of D1-like receptors and the inhibitory influence of D2-like receptors.

### Repeated Application of Dopamine and Dopamine Receptor Agonists

Preliminary experiments showed that application of dopamine caused strong increases or decreases in firing frequency in VP neurons. As the following experiments involved pharmacological protocols with repeated applications of dopamine (or dopamine receptor agonists) in the presence of different ligands, it was important to establish whether neuronal responses to subsequent short applications of dopamine were similar, or whether significant sensitization or desensitization (Chen et al., 1996; Otani et al., 1998; Calabresi et al., 2007) were observed.

**Figure 4:**
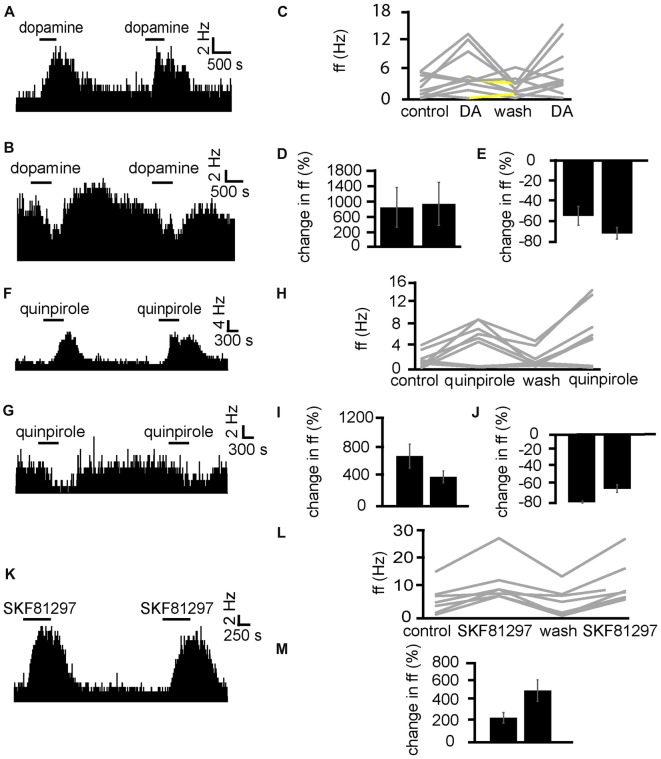
Repeated application of dopamine, D1-like receptor agonists and D2-like receptor agonists produce no clear sensitization effects in VP neurons. **(A)** Excitatory responses to two similar dopamine applications (separated by complete washout) in a VP neuron. **(B)** Inhibitory responses to two dopamine applications in a different VP neuron. **(C)** Changes in firing frequency for 10 VP neurons induced by repeated dopamine applications similar to that illustrated in panels **(A,B)**. Firing frequency was measured before the first application of dopamine, during the first application of dopamine, after dopamine wash out and finally during the second application of dopamine. Responses characterized by significant (*P* < 0.05) changes in firing frequency are in gray, the other ones in yellow. **(D)** Firing frequency during first and second dopamine application for neurons excited by dopamine. **(E)** Firing frequency during first and second dopamine application for neurons inhibited by dopamine. **(F)** Excitatory responses to two quinpirole applications (separated by complete washout) in a VP neuron. **(G)** Inhibitory response to two quinpirole applications in a different neuron. **(H)** Changes in firing frequency for nine VP neurons to the repeated application of quinpirole in experiments similar to that illustrated in panels **(A,B)**. Firing frequency was measured before the first application of quinpirole, during the application of quinpirole, after wash out, before the second application of quinpirole and finally during the second application of quinpirole. Responses characterized by significant (*P* < 0.05) changes in firing frequency are in gray, the other ones in yellow. **(I)** Firing frequency changes during first and second application of quinpirole in neurons excited by quinpirole. **(J)** Firing frequency changes during first and second application of quinpirole in neurons inhibited by quinpirole application. **(K)** Excitatory responses to two applications of SKF81297 (separated by complete washout) in a VP neuron. **(L)** Changes in firing frequency for seven VP neurons in response to repeated SKF81297 application in experiments similar to that illustrated in panel **(A)**. Firing frequency was measured; before the first application of SKF81297, during the application of SKF81297, after wash out, before the second application of SKF81297 and finally during the second application of SKF81297. Responses characterized by significant (*P* < 0.05) changes in firing frequency are in gray, the other ones in yellow. **(M)** Firing frequency changes during first and second application of SKF81297 for neurons excited by SKF81297 application.

Dopamine was applied twice (the second application was carried out after complete washout of the first). In two experiments, 10 VP neurons were identified for analysis, as they responded to dopamine. In seven of these neurons, dopamine significantly (*P* < 0.05) increased firing (Figure 4A), while in the other three dopamine significantly (*P* < 0.05) decreased firing (Figure 4B). In all cases, the second application of dopamine produced similar effects to the first one, as the firing frequencies measured during the first and the second application of dopamine were not significantly different for any of the 10 neurons (Figures 4D,E). We concluded that repeated exposure to dopamine elicited similar effects in VP neurons (Figure 4C).

Having established that repeated application of dopamine caused similar responses in VP neurons, we carried out the same protocol for D1-like and D2-like receptor agonists, to ensure the responses to these agonists did not undergo sensitization or desensitization upon repeated exposure.

Next, quinpirole was applied twice (the second application was carried out after complete washout of the first). In two experiments, nine VP neurons were identified for analysis, as they responded to quinpirole. In six of these neurons, quinpirole significantly (*P* < 0.05) increased firing (Figure 4F), while in the other three quinpirole significantly (*P* < 0.05) decreased firing (Figure 4G). In all cases, the second application of quinpirole produced similar effects to the first one, as the firing frequencies measured during the first and the second application of quinpirole were not significantly different for any of the nine neurons (Figures 4H–J). We concluded that repeated exposure to D2-like receptor agonists elicited similar effects in VP neurons.

Next, SKF81297 was repeatedly applied (with the second application after complete wash out of the first) to test if differences were seen due to repeated exposure (Figure 4K). In two experiments, seven neurons in the VP were identified for analysis as they responded to SKF81297. 7/7 neurons in the VP studied showed a statistically significant (*P* < 0.05) increase in firing frequency in response to SKF81297 application. In all cases, the second application of SKF81297 produced similar effects to the first one, as the firing frequencies measured during the first and the second application of SKF81297 were not significantly different for any of the seven neurons (Figures 4L,M). We concluded that repeated exposure to D1-like receptor agonists elicited similar effects in VP neuron.

### Role of D1-Like and D2-Like Receptors in the Modulation of VP Neurons

To dissect out the modulatory effects of D1-like and D2-like receptors on VP neurons and to confirm the ability to identify the neuron types based upon spike half-width, dopamine was applied in the presence of D1-like and D2-like receptor antagonists.

**Figure 5:**
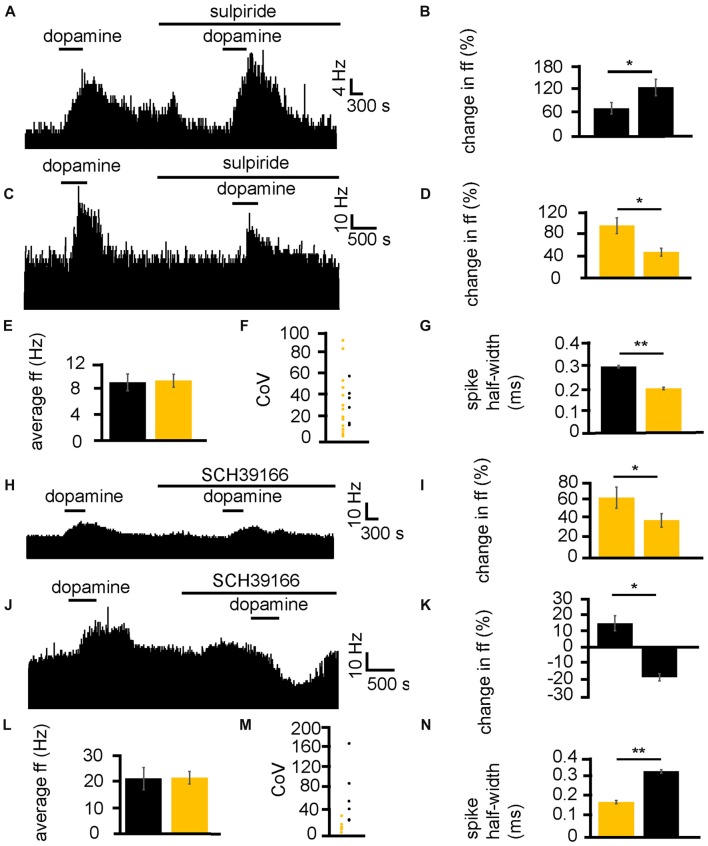
D1-like and D2-like receptor antagonists modulate the effect of dopamine in the VP. **(A)** For some neurons, when dopamine was applied in the presence of sulpiride, it produced a larger increase in firing than when it was applied in the absence of sulpiride. **(B)** Firing frequency changes to dopamine application in control and in the presence of sulpiride, for neurons similar to that illustrated in panel **(A)**. **(C)** For some neurons, when dopamine was applied in the presence of sulpiride, it produced a smaller increase in firing than when it was applied in the absence of sulpiride. **(D)** Firing frequency changes to dopamine application in control and in the presence of sulpiride, for neurons similar to that illustrated in panel **(C)**. **(E)** Firing frequency changes to dopamine application for neurons that showed a larger increase in firing to dopamine application (black) in the presence of sulpiride and neurons that showed a smaller increase in firing to dopamine application in the presence of sulpiride (yellow). **(F)** CoV for neurons that showed a larger increase in firing to dopamine in the presence of sulpiride (black) and those that showed a smaller increase in firing to dopamine in the presence of sulpiride (yellow). **(G)** Significant differences in spike half-width (ms) for neurons that showed a larger increase in firing to dopamine in sulpiride (black) compared to those that showed a smaller increase in firing to dopamine in sulpiride (yellow). **(H)** For some neurons, when dopamine was applied in the presence of SCH39166, it produced a smaller increase in firing than when it was applied in the absence of SCH39166. **(I)** Firing frequency changes during dopamine application and dopamine in the presence of SCH39166, for neurons similar to that illustrated in panel **(H)**. **(J)** Excitatory responses to dopamine application followed (after complete washout of dopamine) by inhibitory responses to dopamine application in the presence of SCH39166. **(K)** Firing frequency changes during application of dopamine and dopamine in the presence of SCH39166, for neurons similar to those illustrated in panel **(J)**. **(L)** Firing frequency changes to dopamine application for those neurons that showed a smaller increase in firing to the application of dopamine in the presence of SCH39166 (yellow), and those that showed a decrease in firing frequency to dopamine in the presence SCH39166 (black). **(M)** CoV for neurons that showed a smaller increase in firing to dopamine in the presence of SCH39166 (yellow), and those that showed a decrease in firing frequency to dopamine in the presence of SCH39166 (black). **(N)** Significant differences in spike half-width (ms) for those neurons that showed a smaller increase in firing to dopamine in the presence of SCH39166 (yellow), compared to those that showed a decrease in firing frequency to dopamine in the presence of SCH39166 (black).

Dopamine was applied twice, first alone and second (after complete washout of the first application) in the presence of the D2-like receptor antagonist sulpiride. In two experiments, 23 VP neurons were identified for analysis, as they responded to dopamine. 16/ 23 of these neurons had a significantly (*P* < 0.05) smaller increase in firing in response to dopamine in the presence of sulpiride than to dopamine alone (Figures 5C,D). On the other hand, 7/23 of these neurons had a significantly (*P* < 0.05) larger increase in firing in response to dopamine in the presence of sulpiride than to dopamine alone (Figures 5A,B).

In order to ascertain if this effect of sulpiride on the excitatory effects of dopamine was related to classes of neurons within the VP, baseline firing frequency (Figure 5E), CoV (Figure 5F) and spike half-width (Figure 5G) were calculated. There was no significant (*P* > 0.05) difference in average baseline firing frequency for those neurons that had a larger increase in firing in response to dopamine in the presence of sulpiride, than those that had a smaller increase in firing in response to dopamine in the presence of sulpiride. There was also no significant (*P* > 0.05) difference in the CoV for those neurons that had a larger increase in firing in response to dopamine in the presence of sulpiride, than those that had a smaller increase in firing in response to dopamine in the presence of sulpiride. On the other hand, there was a significant (*P* < 0.05) difference in the spike half-width for those neurons that had larger increase in firing in response to dopamine in the presence of sulpiride 0.29 ± 0.01 ms, with a range of 0.25–0.34 ms, than those that had a smaller increase in firing in response to dopamine in the presence of sulpiride 0.19 ± 0.01 ms, with a range of 0.13–0.24 ms. These results proved consistent with those seen in Figures 2F–H, 3H.

Next, the same experimental protocol was applied as in Figures 5A,C, but with the D1-like receptor antagonist SCH39166. In two experiments, 11 VP neurons were identified for analysis, as they responded to dopamine. 5/11 of these neurons had a significantly (*P* < 0.05) smaller increase in firing in response to dopamine in the presence of SCH39166 than to dopamine alone (Figures 5H,I). Moreover, 6/11 of these neurons had a significantly (*P* < 0.05) inhibitory response to dopamine in the presence of SCH39166 while being excited by dopamine alone (Figures 5J,K).

In order to ascertain if the responses to dopamine in the presence of SCH39166 were related to different classes of neurons within the VP, baseline firing frequency (Figure 5L), CoV (Figure 5M) and spike half-width (Figure 5N) were calculated.

There was no significant (*P* > 0.05) difference in average baseline firing frequency (Figure 5L) for those neurons that had a smaller increase in firing (yellow) in response to dopamine in the presence of SCH39166 compared to those that displayed an inhibitory response to dopamine in the presence of SCH39166 (black). There was also no significant difference in CoV (Figure 5M) for those neurons that had a smaller increase in firing (yellow) in response to dopamine in the presence of SCH39166 compared to those that displayed an inhibitory response to dopamine in the presence of SCH39166 (black). On the other hand, there was a statistically significant (*P* < 0.05) difference in spike half-width (Figure 5N) between those neurons that had a smaller increase in firing (yellow) in response to dopamine in the presence of SCH39166 0.18 ± 0.01 ms, with a range of 0.12–0.23 ms, compared to those that displayed an inhibitory response to dopamine in the presence of SCH39166 0.34 ± 0.01 ms (black), with a range of 0.28–0.39 ms. Again, these results proved consistent with those seen in Figures 2F–H, 3H.

We can conclude that D2-like receptors are responsible for both excitation and inhibition of VP neurons, and that these responses are in distinct groups of neurons, displaying different spike half-width profiles. We can confirm that these data support the conclusion that those neurons with a longer spike half-width profile are excited by D1-like receptor agonists and inhibited by D2-like receptors agonists, while those with a shorter spike half-width profile are excited by both D1-like and D2-like receptor agonists. Those neurons excited by both D1 and D2 agonists and with a shorter spike half-width profile are referred to as type I neurons, while those neurons inhibited by D2 agonists with a longer spike half-width profile are referred to as type II neurons.

### D1-Like Receptors Exert Their Effects Presynaptically, While D2-Like Receptors Cause Excitation Presynaptically and Inhibition Postsynpatically

Because of the GABAergic and Glutamatergic inputs of the VP (Root et al., 2015) and the number of different interneurons, including cholinergic neurons (Pang et al., 1998; Zaborszky and Duque, 2000). It could be that D1-like receptors have their modulatory effects, presynaptically, postsynaptically or at both points. To identify whether D1-like receptors caused the increase in firing frequency in VP neurons acting pre- or postsynaptically, SKF81297 was applied in a solution of aCSF with low levels of Ca^2+^, used to block presynaptic transmission. This was compared to application of SKF81297 in standard aCSF.

**Figure 6:**
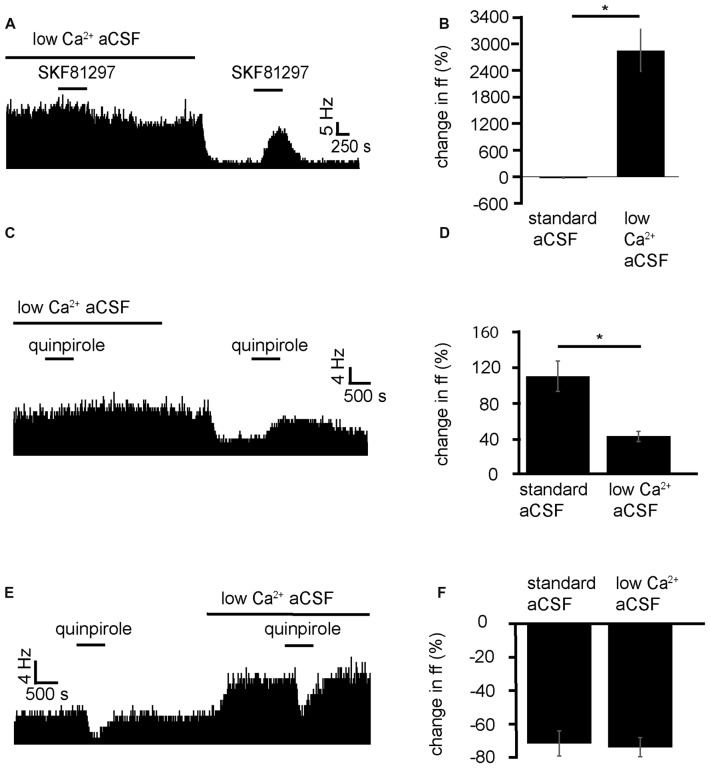
Low Ca^2+^ aCSF blocks the excitatory effects of D1-like and D2-like receptor agonists but not the inhibitory effects of D2-like receptor agonists. **(A)** Excitatory response to SKF81297 application in standard aCSF, which is not present in low Ca^2+^. **(B)** Significant differences in firing frequency for SKF81297 application in low Ca^2+^ compared to the application of SKF81297 in standard aCSF. **(C)** Excitatory responses to quinpirole application alone were occluded in response to quinpirole application in low Ca^2+^ aCSF. **(D)** Significant difference in firing frequency for quinpirole application alone compared to quinpirole application in low Ca^2+^ aCSF. **(E)** Inhibitory responses to quinpirole application persevere to quinpirole application in the presence of low Ca^2+^ aCSF. **(F)** Firing frequency responses to quinpirole alone compared to quinpirole application in low Ca^2+^ aCSF.

SKF81297 was applied twice, first in the presence of low Ca^2+^ aCSF and second (after complete washout of the first) in standard aCSF. In two experiments, 7/7 spontaneously active neurons in the VP showed no significant change in firing frequency in response to SKF81297 in low Ca^2+^ conditions (Figure 6B). However, 5/7 neurons, showed strong significant (*P* < 0.05) increases in excitation 2856 ± 1079% in response to SKF81297 when subsequently applied in standard aCSF (Figure 6B). These five neurons also showed statistically significant (*P* < 0.05) decreases in firing in response to wash-in of standard aCSF after prolonged exposure to low Ca^2+^.

We can therefore conclude that D1-like receptors largely have their effects presynaptically. However, the question remains as to whether this is through disinhibition, facilitation, or through another neurochemical mechanism.

Next the D2-like receptor mediated responses were investigated. D2-like receptor agonists have been shown in Figure 3 to produce both excitation and inhibition in the firing of VP neurons. Mengual and Pickel (2002) have shown that some D2-like receptors are located presynaptically in the VP. Little else is known about their site of action within the VP. It was therefore imperative to investigate this further. To clarify the mechanism of action of D2-like receptors in the VP, and to investigate how this relates to their dichotomous effects on firing frequency, the effects of quinpirole in standard aCSF were compared to those in low Ca^2+^ aCSF, which was used to block presynaptic transmission.

From four experiments, 12 neurons were identified in the VP for analysis that responded to quinpirole application. 8/12 of these neurons responded with significant (*P* < 0.05) increases in firing frequency to the application of quinpirole and 4/12 of these neurons responded with significant (*P* < 0.05) decreases in firing frequency to the application of quinpirole.

Of the eight that were excited by quinpirole application, 6/8 showed non-significant responses to quinipriole application in the presence of low Ca^2+^ aCSF, and 2/8 show significant (*P* < 0.05) increases in firing frequency to quinpirole application in the presence of low Ca^2+^. For these eight neurons, the application of quinpirole alone produced an average percentage increase in firing frequency of 111 ± 34% compared to 42 ± 11% for the application of quinpirole in the presence of low Ca^2+^ (Figure 6D). In 6/8 cases the first application of quinpirole (alone) produced significantly (*P* < 0.05) different responses to the second application (in low Ca^2+^) as the firing frequencies measured during the first (alone) and second (in the presence of low Ca^2+^) application of quinpirole were significantly different (Figure 6D). In 2/8 cases the second application of quinpirole (in low Ca^2+^) produced similar effects to the first one (alone) as the firing frequencies measured during the first and second application of quinpirole, for these two neurons, were not significantly different.

Of the four that were inhibited by quinpirole application all four continued to show significant (*P* < 0.05) decreases in firing frequency to quinpirole application in the presence of low Ca^2+^. For these four neurons, the average decrease in firing frequency was −65 ± 13% in response to quinpirole application alone and −67 ± 10% to quinpirole application in the presence of low Ca^2+^ (Figure 6F). In all four cases, the second application of quinpirole (in low Ca^2+^) produced similar effects to the first one (alone) as the firing frequencies measured during the first and second application of quinpirole were not significantly different (Figure 6F).

We conclude that D2-like receptors mediate an increase in firing frequency, acting presynaptically in the majority of VP neurons, while D2-like receptors mediate a decrease in firing frequency acting postsynaptically in a minority of VP neurons.

### D1-Like Receptors Excite VP Neurons Through Presynaptic Modulation of Glutamate

Because the VP has both GABAergic and glutamatergic inputs (Root et al., 2015), these transmitters were potentially involved in the D1-like receptor agonist action in the VP. As current research suggests that D1 receptors are located presynaptically in the VP, and D1-like receptors are largely considered to be excitatory (Vallone et al., 2000), it seemed possible that they were modulating glutamatergic inputs and therefore increasing neuronal firing in the VP via increased release of GA. To test this hypothesis, SKF81297 was applied twice, first alone and then in the presence of ionotropic glutamate receptor antagonists (NBQX and AP5). The second application was carried out after complete washout of the first.

**Figure 7:**
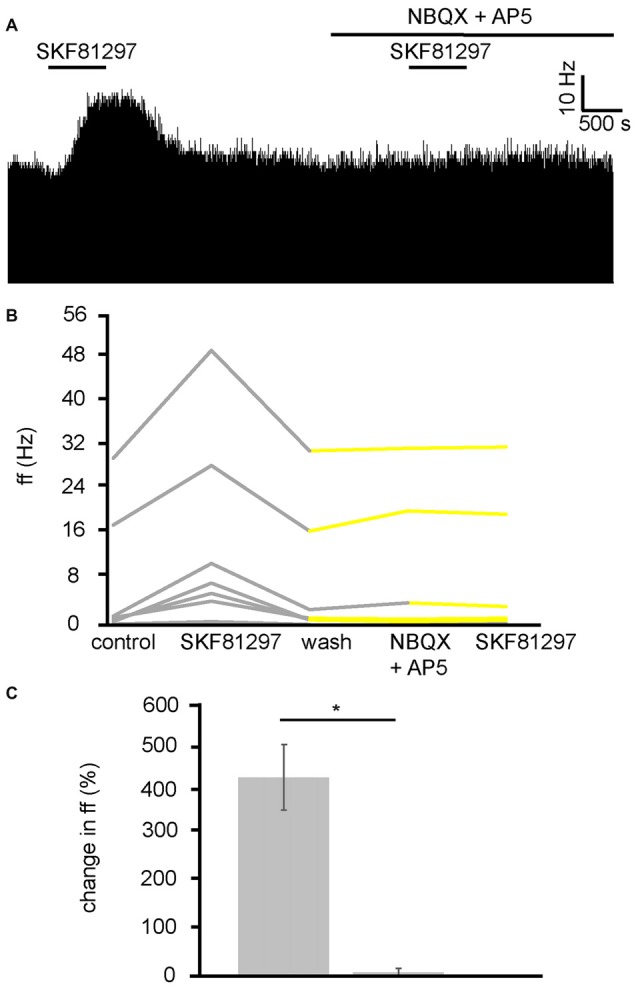
D1-like receptor mediated excitation requires ionotropic glutamate receptors. **(A)** Excitatory responses to SKF81297 application alone that are no longer present in response to SKF81297 in the presence of NBQX and AP5. **(B)** Firing frequency for (ff) seven VP neurons in response to SKF81297 application and SKF81297 application in the presence of NBQX and AP5, similar to that illustrated in panel **(A)**. Firing frequency was measured before the application of SKF81297, during the application of SKF81297, after a period of wash out, in response to the application of NBQX and AP5 and finally in response to SKF81297 in the presence of NBQX and AP5. Responses characterized by significant (*P* < 0.05) changes in firing frequency are in gray, the other ones in yellow. **(C)** Significant differences in firing frequency (ff) to SKF81297 alone compared to the application of SKF81297 in the presence of NBQX and AP5.

In two experiments, seven spontaneously active VP neurons were identified for analysis as they responded to SKF81297. All seven neurons studied in the VP showed strong significant (*P* < 0.05) increases in firing frequency in response to SKF81297 (Figure 7B). However, all seven showed no significant change in firing frequency in response to NBQX and AP5 application, and no significant change in firing frequency in response to subsequent SKF81297 application in the presence of NBQX and AP5 (Figures 7A,B). In all cases the firing frequencies measured during the first and the second application of SKF81297 were significantly (*P* < 0.05) different. The first application of SKF81297 produced an average increase in excitation of 439 ± 144% compared to 6 ± 6% for the second application of SKF81297 (Figure 7C), in the presence of NBQX and AP5.

We can therefore conclude that the increase in firing frequency produced in the VP as a result of D1-like receptor agonists application is a result of facilitation of presynaptic glutamate terminals and the subsequent increase in activation of ionotropic receptors on VP neurons.

### Excitatory Effects of D2-Like Receptor Agonists Require mGluRs

In order to establish the likely neurotransmitter, and receptor group that quinpirole was modulating to increase firing frequency in VP neurons, quinpirole was applied in the presence of MCPG, which is a non-selective mGluR antagonist. Because the current research has identified that quinpirole has its excitatory effects by modulation of presynaptic mechanisms (Figure 6) and D2-like receptors presynaptically are largely considered inhibitory (Vallone et al., 2000; Mengual and Pickel, 2002). Our hypothesis was that D2-like receptors might be modulating glutamate release. Group 2 and 3 mGluRs are known to be inhibitory (Benarroch, 2008) and therefore provide a potential mechanism by which presynaptic inhibition of glutamate by D2-like receptors agonists might disinhibit neurons in the VP and therefore increase firing frequency in a number of VP neurons.

**Figure 8:**
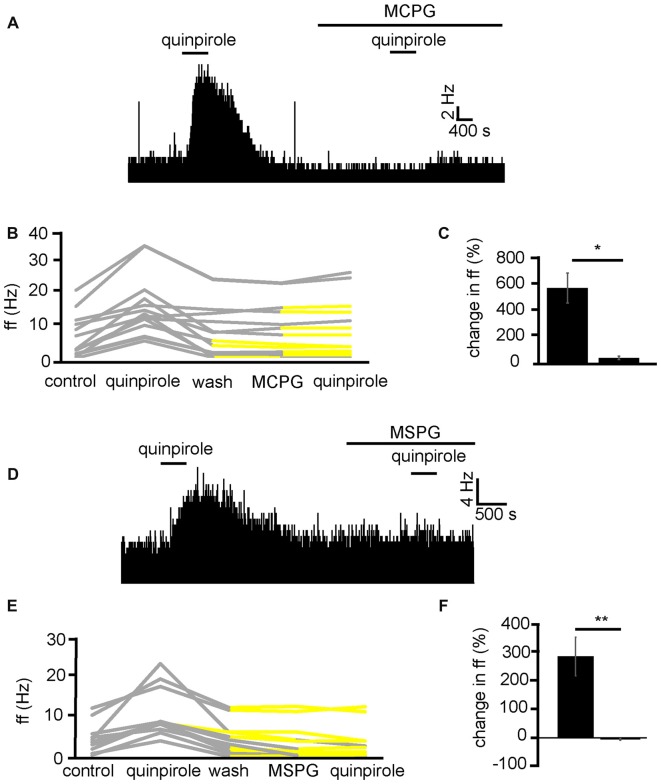
Excitatory effects of D2-like receptor agonists require metabotropic glutamate receptors. **(A)** Excitatory responses to quinpirole that are not present for the same neuron in response to quinpirole in the presence of MCPG. **(B)** Changes in firing frequency for 13 VP neurons to application of quinpirole and quinpirole in the presence of MCPG, similar to that illustrated in panel **(A)**. Firing frequency was measured before the application of quinpirole, during the application of quinpirole, after wash out of quinpirole, during application of MCPG and finally during the application of quinpirole in the presence of MCPG. Responses characterized by significant (*P* < 0.05) changes in firing frequency are in gray, the other ones in yellow. **(C)** Significant differences in firing frequency in response to quinpirole compared to quinpirole in the presence of MCPG. **(D)** Excitatory responses to quinpirole that are not present for the same neuron in response to quinpirole in the presence of MSPG. **(E)** Changes in firing frequency for 11 VP neurons to the application of quinpirole and quinpirole in the presence of MSPG, similar to that illustrated in panel **(A)**. Firing frequency was measured; before the application of quinpirole, during the application of quinpirole, after wash out of quinpirole, during application of MSPG and finally during the application of quinpirole in the presence of MSPG. Responses characterized by significant (*P* < 0.05) changes in firing frequency are in gray, the other ones in yellow. **(F)** Significant (*P* < 0.05) differences in firing frequency in response to quinpirole compared to quinpirole in the presence of MSPG.

In two experiments, 13 VP neurons were identified for analysis as they responded to quinpirole. All 13 neurons studied in the VP showed strong significant (*P* < 0.05) increases in firing frequency in response to quinpirole. After a period of wash out a further significant (*P* < 0.05) change was seen in 9/13 of those neurons studied in response to application of the non-selective mGluR antagonist MCPG. Subsequently only 4/13 of these neurons showed any significant (*P* < 0.05) increase in firing frequency in response to quinpirole in the presence of MCPG (Figure 8B). In the majority of cases, the first application of quinpirole resulted in distinctly different responses to the second, as the firing frequencies measured during the first and the second application of quinpirole were significantly (*P* < 0.05) different for 12/13 neurons (Figure 8A). The first application of quinpirole producing an average percentage increase in excitation of 588 ± 232% compared to 41 ± 27% for the second application of quinpirole, in the presence of MCPG (Figure 8C).

To further explore the role of mGluRs in the excitatory effects of D2-like receptor agonists we applied quinpirole alone and in the presence of MSPG (Figure 8D), which is a selective antagonists for group 2 and 3 mGlu receptors, which are known to have inhibitory effects (Nicoletti et al., 2011) and be located postsynaptically (Petralia et al., 1996).

From three experiments, 11 neurons were identified for analysis as they responded to quinpirole application. All 11 neurons studied in the VP showed strong significant (*P* < 0.05) increase in firing frequency in response to quinpirole. After a period of wash out a further significant (*P* < 0.05) change was seen in 3/11 of those neurons studied in response to MSPG application. Subsequently only 2/11 of these neurons showed any significant (*P* < 0.05) change in firing frequency in response to quinpirole in the presence of MSPG (Figure 8E). In all cases the firing frequencies measured during the first (alone) and second (presence of MSPG) application of quinpirole were significantly (*P* < 0.05) different (Figure 8F).

We therefore concluded that D2-like receptors inhibit the release of glutamate presynaptically, which in turn disinhibits VP neurons, which express inhibitory group 2/3 mGluRs, increasing their firing frequency.

### Inhibitory Effects of D2-Like Receptor Agonists Persevere in the Presence of mGluR Antagonists

While excitation in VP neurons, as a result of quinpirole application, has been shown in the current study to be modulated by presynaptic mechanisms (Figures 6C,D). The current study suggests that inhibition of firing frequency in the minority of VP neurons is likely to be as a result of postsynaptic /direct inhibition (Figures 6E,F). This should mean that MCPG has no effect on the reduction in firing frequency seen in response to quinpirole.

**Figure 9:**
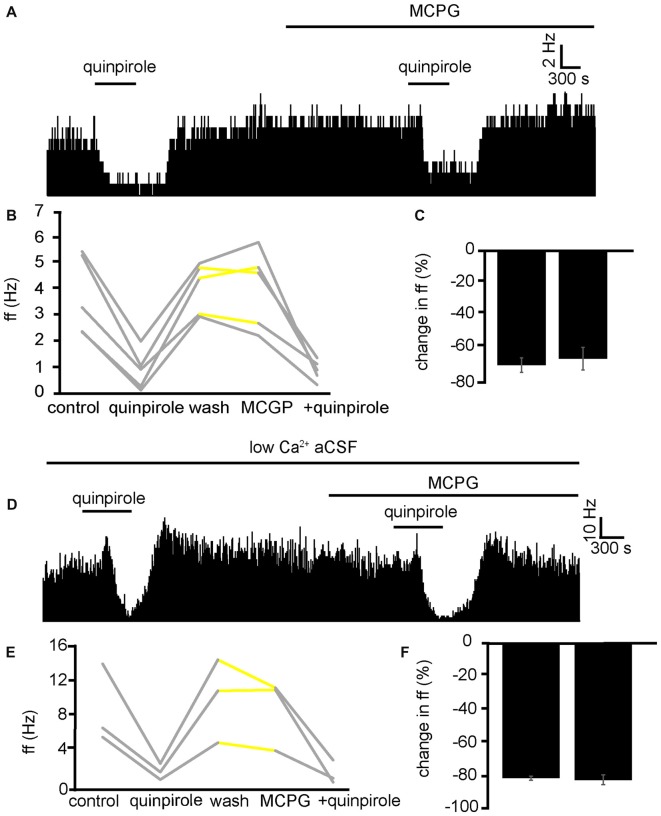
Inhibitory effects of D2-like receptor agonists continue in the presence of mGluR antagonists and low Ca^2+^ aCSF. **(A)** Inhibitory responses to quinpirole application alone and in the presence of MCPG. **(B)** Changes in firing frequency for five VP neurons to application of quinpirole and quinpirole in the presence of MCPG, similar to that illustrated in panel **(A)**. Firing frequency was measured before the application of quinpirole, during the application of quinpirole, after wash out of quinpirole, during application of MCPG and finally during the application of quinpirole in the presence of MCPG. Responses characterized by significant (*P* < 0.05) changes in firing frequency are in gray, the other ones in yellow. **(C)** Firing frequency in response to quinpirole compared to quinpirole in the presence of MCPG. **(D)** Inhibitory responses to quinpirole application in the presence of low Ca^2+^ aCSF are repeated in the presence of low Ca^2+^ aCSF and MCPG. **(E)** Changes in firing frequency for three VP neurons to application of quinpirole and quinpirole in the presence of MCPG, similar to that illustrated in panel **(A)**. Firing frequency was measured; before the application of quinpirole, during the application of quinpirole, after wash out of quinpirole, during application of MCPG and finally during the application of quinpirole in the presence of MCPG. Responses characterized by significant (*P* < 0.05) changes in firing frequency are in gray, the other ones in yellow. **(F)** Firing frequency in response to quinpirole in low Ca^2+^ aCSF compared to quinpirole in the presence of low Ca^2+^ aCSF and MCPG.

In three experiments, five VP neurons were identified for analysis as they responded to quinpirole application. 5/5 of these neurons measured in the VP displayed significant (*P* < 0.01) decreases in firing frequency in response to quinpirole. After a period of wash out MCPG application evoked significant changes in firing frequency in 2/5 neurons, subsequently application of quinpirole in the presence of MCPG evoked a further significant (*P* < 0.05) decrease in firing frequency in 5/5 of these VP neurons (Figure 9A). In the majority of cases, the second application of quinpirole produced similar effects to the first one, as the firing frequencies measured during the first and the second application of quinpirole were not significantly different for 4/5 neurons (Figures 9B,C). The first application of quinpirole producing an average percentage inhibition of −69 ± 8% compared to −66 ± 9% for the second application of quinpirole, in the presence of MCPG (Figure 9C).

To further ensure that the inhibitory effect of D2-like receptor agonists was not mediated presynaptically or through mGlu receptors, we applied quinpirole in the presence of low Ca^2+^ aCSF and in the presence of low Ca^2+^ aCSF and MCPG (Figure 9D).

From one experiment, three neurons were identified for analysis. 3/3 displayed significant (*P* < 0.05) decreases in firing frequency in response to application of quinpirole. After a period of wash out 3/3 of the neurons responded with non-significant (*P* > 0.05) changes in response to MCPG application, subsequent application of the quinpirole in the presence of low Ca^2+^ and MCPG evoked further significant (*P* < 0.05) decreases in firing frequency in 3/3 of the neurons. In all cases, the second application of quinpirole (in the presence of MCPG and low Ca^2+^) produced similar effects to the first one (Figures 9E,F), as the firing frequencies measured during the first and the second application of quinpirole were not significantly different for 3/3 neurons.

We can conclude that mGluR are not involved in the inhibition seen in the VP to quinpirole application.

### Excitatory Responses to D1-Like Receptor Agonists Are Not Effected by mGluR Antagonists

**Figure 10:**
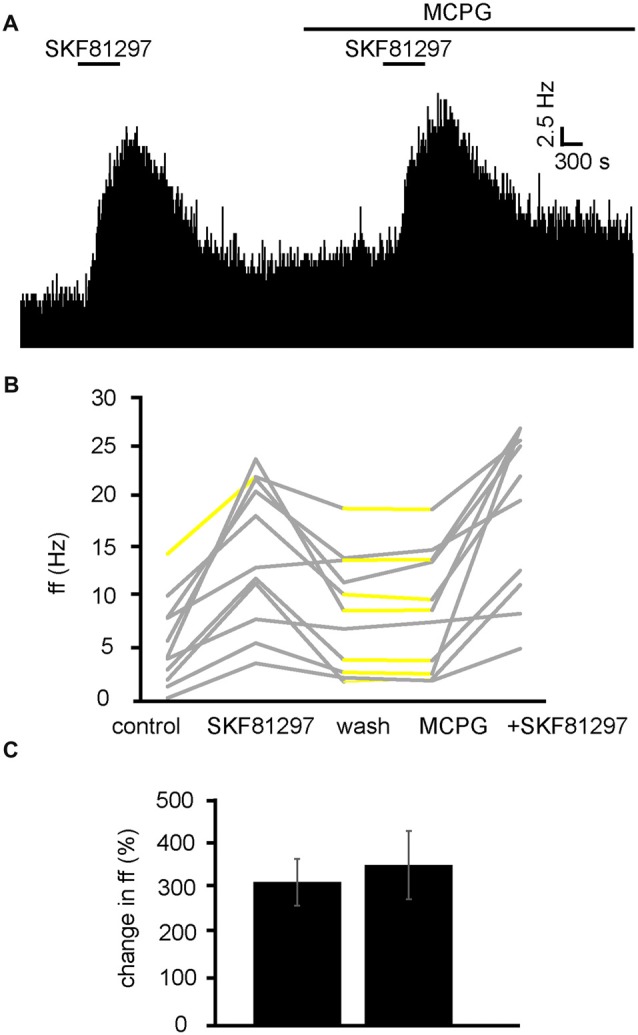
D1-like receptor agonists continue to excite VP neurons in the presence of MCPG. **(A)** Excitatory responses to SKF81297 application alone and in the presence of MCPG. **(B)** Changes in firing frequency for 11 VP neurons to application of SKF81297 and SKF81297 in the presence of MCPG, similar to that illustrated in panel **(A)**. Firing frequency was measured; before the application of SKF81297, during the application of SKF81297, after wash out of SKF81297, during application of MCPG and finally during the application of SKF81297 in the presence of MCPG. Responses characterized by significant (*P* < 0.05) changes in firing frequency are in gray, the other ones in yellow. **(C)** Firing frequency in response to SKF81297 compared to SKF81297 in the presence of MCPG.

We have already established that increases in firing frequency as a result of SKF81297 application are largely modulated by glutamate and ionotropic glutamate receptors. To ensure mGluRs do not interfere with this response, experiments were carried out with SKF81297 in the presence of MCPG (Figure 10A). From 2 experiments, 11 neurons were identified for analysis. 10/11 of these neurons were excited (*P* < 0.05) by SKF81297 application. After a period of wash out 4/11 neurons responded with significant (*P* < 0.05) changes in response to MCPG application, subsequent application of SKF81297, evoked a further significant increase in firing frequency in 11/11 of the VP neurons observed. In the majority of cases, the second application of SKF81297 (in the presence of MCPG) produced similar responses to the first one, as the firing frequencies measured during the first and the second application of SKF81297 were not significantly different for 10/11 neurons (Figures 10B,C). The first application of SKF81297 producing an average percentage excitation of 322 ± 105% compared to 361 ± 153% for the second application of SKF81297, in the presence of MCPG (Figure 10C).

We can therefore rule out the involvement of mGluRs in the excitation observed in response to SKF81297 in the VP.

## Discussion

This study investigated the effects of dopamine on the tonic firing of VP neurons *in vitro*. Our data shows that dopamine has dual effects in the VP, producing increases and decreases in firing frequency in VP neurons. Two populations of neurons in the VP were identified based upon their spike half-width profile. Type I neurons displaying a markedly shorter spike half-width profile and being excited by both D1-like and D2-like receptor agonists. This resulted in a net excitatory effect in response to dopamine in type I neurons. Type II neurons however had a markedly longer spike half-width profile, were inhibited by D2-like agonists and excited by D1-like agonists. For type II neurons the net balance of inhibitory D2-like receptor activation and excitatory D1-like receptor activation, through application of dopamine, resulted in a net effect of increased firing rates in some type II neurons and decreased firing rates in others.

### Presynaptic and Postsynaptic Effects of Dopamine

Previous studies of the dopaminergic modulation of VP neurons had been carried out *in vivo*. These studies showed that dopamine can exert both excitatory and inhibitory influences on VP neurons. Electrical stimulation of the VTA or SNc caused inhibitions or excitations of VP neurons, in a roughly 2:1 ratio (Napier and Maslowski-Cobuzzi, 1994; Mitrovic and Napier, 2002). Similar responses were observed with local microiontophoretic applications of dopamine (Napier and Potter, 1989; Mitrovic and Napier, 2002). Dopamine D1-like and D2-like receptors are thought to contribute to these responses, however it is unclear, which modulate excitations and which modulate inhibitions. Maslowski and Napier (1991a) found D2-like receptor agonists reduced activity in 59% of neurons studied and that D1-like receptor agonists excited 69% of neurons. However, Napier and Maslowski-Cobuzzi (1994) data suggest that D1-like receptor agonists largely produce inhibitions in VP neurons and D2-like receptor agonist largely produce excitation.

Both direct and indirect effects of dopamine are considered likely involved (Root et al., 2015) in modulation of VP neurons. However, a dissection of these effects and of the other neurotransmitters involved had not been possible in *in vivo* experiments. Our data clearly indicated that D1-like receptors acted at a presynaptic level, as a D1-like receptor agonist produced no significant effects in low Ca^2+^ (a condition in which synaptic transmission is blocked), even in neurons in which D1-like receptor agonist had previously elicited strong excitatory effects when applied in normal Ca^2+^. Furthermore, we were able to show that D1-like receptors exerted their presynaptic effects by increasing ionotropic glutamate receptors activation, as antagonists of ionotropic glutamate receptors (NBQX and AP5) also consistently blocked the excitatory effects of D1-like receptor agonists. Thus, a facilitation of glutamate release from terminals impinging on VP neurons appears to underlie the excitatory effects of D1-like receptors in both groups of VP neurons.

On the other hand, D2-like receptors engaged both presynaptic and postsynaptic mechanisms. Low Ca^2+^ failed to block the decreases in firing frequency observed in the neurons of the second group in response to D2-like receptor agonist application. Thus, D2-like receptors caused their inhibitory effects acting postsynaptically on VP neurons of the second group. However, the excitatory effects of a D2-like receptor agonist were no longer observed (in neurons of the first group) in low Ca^2+^ aCSF, showing that these effects were mediated presynaptically. A study by Mengual and Pickel (2002) showed that D2-like receptors are located at presynaptic locations on GABAergic terminals in the VP. However, the excitatory effects of D2-like receptor agonists persisted in the presence of GABA_A_ and GABA_B_ receptor antagonists, showing that they did not result from inhibition of GABA release. On the other hand, a broad-spectrum mGluR antagonist completely prevented the excitatory effects of D2-like receptor agonists. Furthermore, a ligand that selectively blocks group 1 and group 2 mGluRs also prevented any excitatory action of D2-like receptor agonists. These observations suggested that the excitatory effects of D2-like receptors were caused by inhibition of glutamate release and reduced postsynaptic activation of inhibitory mGluRs. mGluRs are present in the VP (Testa et al., 1994) and some mGluR groups are inhibitory (Anwyl, 1999). There is a paucity of data on mGluR action within the VP, but Ohishi et al. (1993) data suggests there is labeling for multiple mGluR in the VP, including the inhibitory mGluR2 group. They are also a likely candidate as Holmes et al. (1996) found mGluR2 receptors to be the mediator of postsynaptic hyperpolarizations within the basal lateral amygdala.

**Figure 11:**
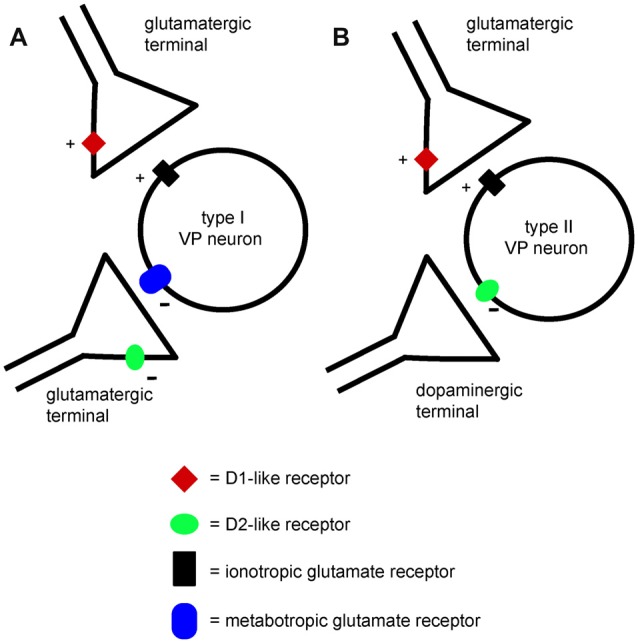
A minimal model explaining the differential effects of D1-like and D2-like receptor activation on type I and type II VP neurons in the VP. **(A)** Presynaptic D1-like receptors facilitate glutamate release and excite type I VP neurons through increased activation of ionotropic glutamate receptors. Another glutamatergic input to type I neurons activates inhibitory metabotropic glutamate receptors. Presynaptic D2-like receptors have an inhibitory effect on this glutamate input, reducing glutamate release and disinhibiting type I neurons. Thus, both D1-like and D2-like receptor have excitatory influences on type I neurons. **(B)** Presynaptic D1-like receptors facilitate glutamate release and excite type II VP neurons by increasing activation of ionotropic glutamate receptors. Postsynaptic D2-like receptors have direct inhibitory effects on type II VP neurons. The net influence of dopamine on type II neurons results from the balance of excitatory D1-like receptor effects and inhibitory D2-like receptor effects.

The observation that NBQX and AP5 did not affect VP neurons firing but prevented the excitatory effects of D1 agonists, suggests that the glutamate terminals facilitated by D1 receptors were not spontaneously active in brain slices. We tentatively propose that a different set of glutamate terminals was responsible for the activation of postsynaptic inhibitory mGluRs in those VP neurons in which mGluR antagonists prevented inhibition by D2 agonists (as illustrated in Figure 11). These terminals are expected to release glutamate spontaneously, keeping postsynaptic inhibitory group 2 and/or 3 mGluRs in a state of basal activation. However, the lack of effects of mGluR antagonists on the firing frequency of these VP neurons was unexpected. It is possible that the excitatory influence caused by reduced activity of mGluRs was compensated by an inhibitory influence caused by reduced activity of other pre or postsynaptic mGluRs affecting VP neurons through different mechanisms. Further experiments will be needed to test this hypothesis.

### Identity of VP Neurons Modulated by Dopamine

As mentioned above, VP neurons displayed two distinct responses to D1-like and D2-like receptor agonists. Type I neurons responded with increases in firing frequency to both specific D1-like and D2-like receptor agonists, while type II neurons were excited by D1-like receptor agonists and inhibited by D2-like receptor agonist. These responses can be attributed unambiguously to two distinct neuronal populations based on their spike half-width profile, with the type I neurons having a shorter spike half-width than type II neurons.

Further supporting this distinction, neurons with a shorter spike half-width displayed a decreased increase in firing frequency to dopamine in the presence of either D1-like or D2-like receptor antagonists, suggesting that both receptor classes contributed to increases in firing frequency for these neurons. Conversely, neurons with a longer spike half-width showed an increased excitatory response to dopamine in the presence of D2-like receptor antagonists and were inhibited by the application of dopamine in the presence of D1-like receptor antagonists, confirming that D2-like receptors inhibit these neurons.

Several studies have investigated spike half-width profiles in the VP and its dorsal extent, the GPe (Bengtson and Osborne, 2000; Bengtson et al., 2004; Abdi et al., 2015; Hernández et al., 2015). VP neurons include prominent cholinergic and GABAergic populations (Zaborszky and Duque, 2000; Duque et al., 2007; Root et al., 2015). Cholinergic cells have sparse axons and project to the cortex and the amygdala (Zaborszky et al., 1986; Carlsen et al., 2004), while GABAergic neurons can have dense axonal arborization and are thought to include both interneurons and projection neurons (Zaborszky et al., 2012). The electrophysiological features of these neuronal types have not been completely identified. Bengtson and Osborne (2000) found that only 2/13 cholinergic neurons in the VP had any tonic firing activity. Bugaysen et al. (2010) suggested that it is impossible to separate neuron populations from extracellular recordings in the rat GPe based on action potential half-width, although Becchetti et al. (2012) suggests that spike half-width maybe a viable way of distinguishing inhibitory from excitatory neurons in mature animals using MEA data.

Pang et al. (1998) identified “Type I” neurons *in vivo*, with features resembling those of noncholinergic neurons described by Bengtson and Osborne (2000). Type B neurons of Lavin and Grace (1996) also have features similar to these cells, and have short spike duration (approximately 1.3 ms). These neurons are probably GABAergic projection neurons (Root et al., 2015). Conversely, “Type II” neurons of Pang et al. (1998) and “Type A” neurons of Lavin and Grace (1996) had relatively similar features, including slower spikes (approximately 2.8 ms). Pang et al. (1998) identified these neurons as noncholinergic (possibly GABAergic) interneurons. Based on this complex picture, we can tentatively classify our type I neurons as GABAergic projection neurons corresponding to type I of Pang et al. (1998) and type B of Lavin and Grace (1996) and our type II neurons as GABAergic interneurons corresponding to type II of Pang et al. (1998) and type A of Lavin and Grace (1996). Further investigation is needed to confirm the neurochemical and morphological identity of the two groups of neurons identified by this study based on their dopaminergic responses.

In some previous *in vivo* experiments dopamine inhibitions of VP neurons were more frequent than dopamine excitations (Napier and Maslowski-Cobuzzi, 1994), while we found a prevalence of excitations. It is likely that this discrepancy results from the fact that in the intact brain the VP receives a number of external inputs (including glutamate) that are silent in brain slices and could be modulated presynaptically by dopamine.

### Functional Implications

The VP is crucially involved in reward processing (Smith et al., 2009) and drug-seeking behavior (Kalivas and Volkow, 2005; Prasad and McNally, 2016). Dopamine levels rise dramatically within the VP as a result of sensitization to drugs of abuse (Stout et al., 2016) and dopaminergic mechanisms in the VP underlie morphine-induced conditioned place preferences (Gong et al., 1996; Zarrindast et al., 2007). The current study provides novel and clear insights into the cellular mechanisms by which dopamine modulate neuronal activity in the VP. Given the prominence of the VP in the reward circuits, understanding how dopamine works in this area is of primary theoretical value, and can also help developing rational pharmacological approaches to fight addiction and other dysfunctions of the reward system.

## Author Contributions

MC designed and carried out experiments, analyzed the data and contributed to writing the manuscript. EB designed the experiments, analyzed the data and contributed to writing the manuscript.

## Conflict of Interest Statement

The authors declare that the research was conducted in the absence of any commercial or financial relationships that could be construed as a potential conflict of interest.
